# Analysis of management decisions and outcomes of a weekly multidisciplinary pediatric tumor board meeting in Uganda

**DOI:** 10.2144/fsoa-2019-0070

**Published:** 2019-09-19

**Authors:** Paul E George, Geriga Fahdil, Israel Luutu, Alfred Bulamu, John Sekabira, Nasser Kakembo, Susan Nabadda, Sam Kalungi, Joyce B Kambugu

**Affiliations:** 1 Baylor College of Medicine, Texas Children's Hospital, Houston, TX 77034, USA; 2 Department of Pediatrics, Uganda Cancer Institute, Kampala, Uganda; 3 Mulago National Referral Hospital, Kampala, Uganda

**Keywords:** Africa, multi-disciplinary tumor board, pediatric, Uganda

## Abstract

**Aim:**

To evaluate the efficacy of a pediatric multidisciplinary tumor board (MTB) in Uganda.

**Patients & methods:**

We documented the discussion of cases presented at a pediatric MTB and subsequently, though retrospective chart review, determined the degree to which decision were implemented.

**Results:**

95 patients were discussed. In total, 129 of 226 (57%) distinct management decisions reached during the MTBs were implemented. Of these, 15 resulted in changes in diagnosis and 53 were classified as major changes in management. Decisions on chemotherapy were the most likely to be successfully enacted (51/58), followed by radiotherapy (18/30) and surgery (12/21). Labs/consults were less likely to be implemented.

**Conclusion:**

Key improvements, specifically in the documentation and implementation of management decisions, are needed to improve the MTB's efficacy.

## Lay abstract

Tumor board meetings, which are standard of care for cancer management and well-studied in high income settings, have not been appropriately evaluated in low-income settings. Therefore, we undertook a review of a weekly pediatric tumor board meeting in Uganda, in which we documented the patients discussed and management decisions reached. We found that many diverse patients were discussed in tumor board meetings, with important implications in diagnosis and management. However, many of the decisions reached in the meetings were not subsequently implemented, underscoring the need to improve the effectiveness of the tumor board meeting.

Two-hundred thousand children and adolescents are diagnosed with cancer annually. Of those, 80% live in low- and middle-income countries (LMICs), where survival of childhood cancer is less than 20% [1]. Disparately, high-income countries (HICs) report survival rates in pediatric cancers above 80% [2]. There are a variety of well-reported reasons for the significantly higher mortality in LMIC, including late presentation and under diagnosis, high-abandonment rates, high prevalence of malnutrition and other comorbidities, suboptimal supportive care and limited access to curative therapies [1,3–8]. Less well-reported is the paucity of multidisciplinary tumor board (MTBs) meetings in LMICs and the effect this has on patient outcomes [9].

MTBs represent best practice for pediatric cancer programs [10]. The crucial role of the MTBs in engendering individualized multidisciplinary patient review, improving accurate diagnoses, enhancing timely appropriate treatment and fostering learning has been well-documented in HICs [11]. Given these inherent attributes of MTBs, it is likely they would play an important role in the management of pediatric cancer in LMICs; however, their role, effectiveness and challenges in such settings have not been documented.

Given the significant survival disparity in children with cancer between LMICs and HICs, it is imperative that every avenue for improving outcomes in children with cancer and reducing this disparity be explored and evaluated. Further, many specialist hours, a particularly scarce resource in developing countries, are accounted for by MTBs, both around the world and in our own institution. Therefore, we have undertaken a review of the weekly pediatric MTB at the Uganda Cancer Institute (UCI) to assess the characteristics and effectiveness of the MTB. Specifically, the objectives of the study are to:

- Determine the number and characteristics of patients discussed at the weekly pediatric MTB over a 6-month time period;
- Document the number, type and importance of management decisions reached at these meetings; and
- Evaluate the degree to which these decisions were enacted.

## Patients & methods

The UCI is an adult and pediatric cancer center, located on the grounds of the Mulago National Referral Hospital in Kampala, Uganda. As the main national referral center for oncologic diagnoses in a country with a population of 44 million people, a wide variety of diseases and presentations present or are referred to the UCI. In total, approximately 500 new patients under the age of 15 years are seen each year, with the most common diagnoses being rhabdomyosarcoma, Wilms tumor, leukemia and lymphoma, often Burkitt lymphoma.

In 2012, the first pediatric MTB at the UCI was formed. This MTB included a pediatric oncologist, a pediatrician, two pathologists, one pediatric surgeon and one radiation oncologist, who met once per week to discuss difficult cases that required multidisciplinary management. Since then, the MTB has evolved and expanded. Currently, there are two weekly pediatric MTBs at the UCI, one for leukemia/lymphoma patients and the other for solid tumor patients. This study focuses exclusively on the solid tumor MTB.

The solid tumor MTB is scheduled for Tuesday mornings and has duration of 90 minutes. Regular conference attendees include members of the following departments: pediatric oncology (chaired by a member of the pediatric oncology service), surgery and pathology. Radiation oncology and radiology intermittently attend. Students and trainees of each department, including residents and fellows, are often present as well. Nurses, patients and/or caretakers are not present. A brief history, current status and relevant labs, imaging and pathology, and question(s) for the MTB are presented by the oncology team for each patient. The case is then discussed, with the MTB deciding on final management recommendations. The primary oncology team, consisting of pediatric hematologists–oncologists, pediatric hematology–oncology fellows and medical officers, are responsible for the ongoing care of the patients.

Beginning in April 2018, a member of the pediatric oncology team documented the MTB recommendations for each patient. These decisions were also recorded in the patients’ paper charts (at the time of the study, patients did not have electronic medical records). The study was a retrospective review of MTB records from May 2018 through October 2018 and a chart review of all patients who had been presented at the weekly MTB during that time frame. Sample size was determined to be each patient presented within the given time frame. Pre-established criteria for inclusion in the study were patients who were presented within the above time frame at the MTB. Patients were excluded if they received their care at other institutions or were presented at the MTB as a consult and/or second opinion.

First, the MTB records were reviewed to determine the patients scheduled and the patients discussed at the MTBs within the time frame. Using the MTB records and confirming with patient charts, the patients’ diagnoses and management plans/medical decisions as proposed at the MTB were recorded. When the MTB discussion resulted in the change of a diagnosis, this event was recorded. Of note, only changes in cancer diagnosis, not changes in disease extent, were coded as changes in diagnosis. Next, each plan/decision was coded in one of the following categories: chemotherapy, surgery, radiation therapy, consult, lab/imaging and other. Major changes in management were also recorded (Table 1).

**Table 1.** Management plan/medical decision category definitions.

Chemotherapy	If a chemotherapy regimen was started, stopped or altered during the MTB, this event was coded as a chemotherapy decision. Major changes in management were coded as those decisions which were not part of routine care or protocol. For example, a patient discussed at the MTB with a new diagnosis of Wilms tumor starting first-line therapy would be coded as a chemotherapy decision, but not a major change. A patient with Wilms tumor found to be progressing on first-line therapy and switched to second-line therapy as a result of the MTB would be coded as a chemotherapy decision and a major change
Surgery	If a surgery was planned during the MTB, this event was coded as a surgical decision. Major changes were coded as those surgeries not part of routine/standard care or protocol. For example, a patient with newly diagnosed Wilms tumor who was scheduled for nephrectomy would be coded as a surgical decision, but not a major change as nephrectomy is standard management for Wilms tumor. A patient with an unknown cystic tumor of the kidney scheduled for nephrectomy would be coded as a surgical decision and major change
Radiation therapy	If radiation therapy was planned during the MTB, this event was coded as a radiation therapy decision. Major changes were coded as those decisions not part of routine/standard care or protocol
Consult	If consultation of another service not represented at the MTB (such as cardiology, nephrology and international consults) was decided during the MTB, this event was coded as a consult decision. Consults were not coded as major changes
Labs/imaging	If a lab or imaging request was recommended by the MTB beyond those labs/images collected as part of routine care, this event was coded as a lab/imaging decision. Labs/imaging decisions were not coded as major changes
Other	Any significant decision that did not reach fit into any of the above categories were coded as Other. Examples of such decisions include: deciding to present again at future MTB, sending samples to outside laboratories, continue active monitoring of the patient, review existing literature related to patient and decide treatment appropriately

MTB: Multidisciplinary tumor board meeting.

Next, a chart review of each patient scheduled and discussed at the meeting was undertaken. Using the patients’ charts and the MTB records, the following data was collected: age, gender, diagnosis, tumor board diagnosis at the time of the MTB, management plan(s) as decided during the MTB (see above) and whether the management plan was enacted. Documentation beyond the patients’ individual paper charts were not pursued (i.e., if a result, outcome or event was not documented in the patient's chart, the decision was coded as not implemented).

This study was approved by the institutional review board at the UCI and informed consent was granted by study subjects/caregivers.

## Results

From May–October 2018, 17 weekly MTBs were held. 183 cases (representing 111 unique patients) were scheduled for presentation during these 17 meetings. In total, 121 cases (95 patients) were discussed in the MTBs, for an average of 7 cases discussed per 90-min MTB. Two patients with missing paper charts (2 cases) were excluded from the analysis, leaving 119 cases (93 patients) included in the final analysis.

The cases analyzed include 53 males and 67 females, with an average age of 6.2 years (range: 6 months to 17 years). Wilms tumor (34 cases, 30 patients) was the most commonly discussed cancer, followed by germ cell tumors, rhabdomyosarcoma and primary central nervous system tumors (Table 2). Totally, 226 distinct management decisions were reached during the MTBs, of which 129 were subsequently implemented (Table 3). Of these decisions, 15 resulted in changes in diagnosis and 52 were classified as major changes in management. Decisions on chemotherapy were the most likely to be successfully enacted (51/58), followed by radiotherapy (18/30) and surgery (12/21). Consults and laboratory/imaging recommendations were less likely to be carried out. Of note, there were often significant delays in management, with 29 of 129 of successfully implemented decisions being enacted with a delay of 4 weeks or greater.

**Table 2.** Diagnoses of patients presented at the multidisciplinary tumor board meeting.

Diagnosis	Cases	Patients
Wilms tumor	34	30
Germ cell tumor	13	8
Rhabdomyosarcoma	12	9
Primary central nervous system	10	7
Neuroblastoma	5	5
Lymphoma	5	5
Retinoblastoma	4	3
Sarcoma (non-rhabdomyosarcoma)^†^	4	3
Carcinoma^‡^	2	2
Hepatoblastoma	1	1
Rhabdoid tumor of the kidney	1	1
Unknown	22	14
Benign	8	6
Total	121	94^§^

† Desmoplastic round blue cell tumor, osteosarcoma and high grade sarcoma.

‡ Mucoepidermoid carcinoma (salivary gland) and nasopharyngeal carcinoma.

§ One patient had two diagnoses (salivary gland adenoma + lymphoma).

**Table 3.** Management decisions reached by the multidisciplinary tumor board and enacted by clinical team.

Management decision	Decisions reached	Decisions enacted	Major decisions reached	Major decisions enacted
Chemotherapy	58	51	21	18
Surgery	21	12	8	4
Radiation therapy	30	18	10	6
Consults	40	16	N/A	N/A
Labs/imaging	53	21	N/A	N/A
Other	24	11	13	6
Total	226	129	52	34

## Discussion

The results of this analysis showed a robust MTB in an African setting, with 121 cases representing 95 unique patients presented in a 6-month period. The MTB resulted in 226 distinct management decisions, with 52 classified as major changes and 15 changes in diagnosis. However, the results also showed poor implementation of management decisions, with only 57% of those management decisions subsequently implemented.

MTBs are ubiquitous in North America and Europe; in fact, they are required for the management of cancer patients in the UK [11] and for accreditation in the USA [10]. MTBs have spread worldwide, including Asia [12], India [13], Australia [14], the Middle East [15,16] and Africa [17]. Literature on the efficacy of MTBs with regards to clinical outcomes has been mixed. For example, studies have shown improvements in diagnosis [18], better adherence to guidelines [19], and a few studies have even shown increased survival in patients discussed at MTBs [20,21]. Conversely, other large studies have shown no differences in outcomes as a result of MTBs [22]. Notable among outcomes research with respect to MTBs, however, is the difficulty in assessing their efficacy. Cancer therapies, protocols and supportive care rapidly evolve. Patient preferences can differ significantly. Given that standard of care involved MTBs in many settings, there is often no control group that is not evaluated by an MTB.

Thus, despite the paucity of hard data showing improvements in the clinical course, MTBs are widely recognized as an integral part of the management of cancer. In one large survey of over 2000 providers, 90% of respondents stated that effective MTBs improved clinical care [11]. Further, by allowing multiple different team members to meet in a structured format, MTBs should improve coordination, communication among many disciplines and save time in patient care [23,24]. However, the research in MTBs comes from settings much different in many aspects than sub-Saharan Africa; specifically, developed countries with more resources, different patient populations and dissimilar cultural attitudes. As such, it is imperative to evaluate MTBs in many different contexts, including the African setting.

In our study, we used implementation as a proxy for the effectiveness of the MTB, a measure which has been recommended for and used as a proxy for effectiveness of MTBs in other settings [25–28]. With only 57% (129/226) of total management decisions and 65% (34/52) of major decisions implemented, our study showed that the implementation of MTB recommendations was quite poor and below the range in other published studies. A systematic review published of MTBs found that previous reports have shown implementation ranging from 84 to 99%, well above our outcome [27]. More recently, a study by Raine *et al.* found that 78% of patients had their MTB plans successfully implemented [28]. Their analysis further showed that difficulty with implementation commonly arose from patient or family choice and difficulties in engaging patients with the service. Further, patients from more deprived areas were less likely to have their treatment plans implemented. A similar study from the UK on implementation of MTB decisions found that of 273 decisions studied, 41 (15%) were not implemented [25]. The most common reasons for non-implementation were co-morbid health issues that had not been discussed sufficiently at the MTB, the influence of patients’ treatment preferences and subsequent clinical information.

Our study did not address the reasons for non-implementation, as such information was not available. However, several conclusions and assumptions can be drawn from the data. At 88%, chemotherapy decisions were the most likely to be enacted. For one, chemotherapy protocols are determined by the oncology team with the availability of chemotherapy agents in mind, so these medicines are typically available for patients. Surgery (57% implementation) and radiation therapy (60% implementation) stand in stark contrast, however, as surgeons, anesthesiologists, operating room time, radiation oncologists and radiotherapy machine time are all limited resources in our setting. However, there are possible reasons beyond resources which may explain the disparity in implementation between treatment modalities. The MTB at the UCI is chaired by and has most representation from the oncology team. Also, the oncology team typically has the most interaction and familiarity with the patients. In contrast, there is no anesthesia representation and the surgeons have often not met the patient prior to MTB presentation. Further, radiology and radiation therapy attendance in MTBs is sporadic given a shortage of local staffing. These factors could explain the disparity and highlight the importance of meeting preparation and attendance.

There are areas for improvement in MTB decision implementation within the primary oncology team, as consults/labs/imaging/others were implemented at 41%. No formal documentation or follow-up mechanisms for MTB decisions are in place, which have been discussed as important features of MTBs [11]. Nursing, which is widely considered to be an integral component of these meetings, is notably not typically represented at the UCI pediatric MTB. Unlike surgeons and oncologists who tend to make their decisions based almost solely on biomedical information presented, nurses are more likely to take into account patient preferences and clinical condition, resulting in decisions that are more clinically appropriate and acceptable to patients [29]. Further, reports indicate that the presence of nursing is associated with increased MTB effectiveness [30].

Two other potential area of improvement for the MTB involve patient selection. One, there is no formal or specific criteria for choosing patients to discuss at MTB. Instead, patients are chosen for inclusion by team members who evaluate the patients, such as those at baseline, those meeting important milestones, and most commonly those with distinct clinical challenges. Reviews of MTBs have recommended guidelines, often with specific outlines and/or forms, for choosing and presenting patients [11]. Further, there is no formal triage among patients to be presented at the MTB. Given the high-patient load and limited time, at least 16 patients scheduled for MTB in the 6-month period were left undiscussed. One potential solution offered is that straight-forward patients, those who fit well into existing treatment protocols, be briefly presented for ratification of the treatment plan rather than a complete discussion [31].

Despite the challenges, the pediatric MTB at the UCI has had remarkable successes. Since its inception, the MTB has grown and now involves a diverse team, including most relevant departments and department heads. Part of this success can be attributed to the fact that the MTB represents protected time for most attendees – in fact, lack of protected time has been frequently cited as a key shortcoming in other settings [23]. Dissent and differences of opinions are encouraged in the MTB, which is an important attribute that allows for critical discussion of complex patients and avoidance of group think. The benefits of the MTB extend beyond patient care, as questioning and case discussion benefit the many trainees in attendance. Such learning also extends to specialists finished with their formal training, as different specialties can learn from each other, enhancing both knowledge and professional development. Further, MTBs are important tools for advancing and improving a research agenda. With 121 separate cases representing 93 patients discussed in a 6-month time period, the MTB, despite the troubles with implementation, has solidified itself as an important aspect of pediatric cancer management at the UCI.

There are several important limitations that deserve mention. First, the study's main measure, implementation of MTB decisions, is not a perfect proxy for effectiveness of an MTB, despite its wide use. We cannot say that the decisions made in the MTB were the ‘correct’ decisions for the patients. Second, the data were limited by record keeping; if management of a patient was not recorded, it was coded as not implemented. This method is consistent with other studies, but may miss management that was enacted but not formally recorded [28]. However, the standard practice at the UCI is that patient care events should be recorded, which minimizes this bias. The scope of the study also represents an important limitation; we did not evaluate clinical outcomes and we did not collect information on why plans were not implemented.

## Conclusion

Both shortcomings, specifically the degree to which decisions at a pediatric MTB in Uganda were enacted, and success of the evaluation of a large number of complex patients by a specialized, multidisciplinary team were presented. Potential means of improvement in implementation were presented and future studies could determine if application of such ideas could improve the efficacy of an MTB in an African setting.

## Future perspective

Given their documented efficacy in other settings, MTBs will continue to remain a key aspect of pediatric cancer care, both in high- and low-income settings. However, it will remain imperative for low-income settings to document their successes and failures, as the underlying factors that contribute to the efficacy of MTBs, such as healthcare provider availability, resource constraints and cultural considerations, differ and thus the best practices for MTB implementation will vary. Pediatric oncology programs in low-income settings should therefore monitor outcomes and implement changes to improve the effectiveness of MTBs.

**Summary points**

**Pediatric cancer in low income settings**

- In low income countries, overall survival of childhood cancer is less than 20%; disparately, high income countries (HICs) report survival rates in pediatric cancers above 80%.
- Multi-disciplinary tumor board meetings (MTBs) represent best practices for care of pediatric cancer. However, their role, effectiveness, and challenges in such settings have not been documented.

**Objectives and methods of the current study**

- We have undertaken a review of the weekly pediatric MTB at the Uganda Cancer Institute (UCI) to assess the characteristics and effectiveness of the MTB.
- The solid tumor MTB is a weekly, 90-minute meeting with representatives from subspecialty departments within pediatric oncology, surgery, and radiology.
- From May–October 2018, we documented the discussion and proposed plan of every case presented at a weekly MTB at the Uganda Cancer Institute.
- Through retrospective chart review, our goal was to determine the number and characteristics of patients discussed, management decisions reached, and the degree to which these decisions were implemented.

**Characteristics of the weekly pediatric solid tumor MTB**

- From May–October 2018, 17 weekly MTBs were held.
- 183 cases (representing 111 unique patients) were scheduled, of which 121 cases (95 patients) were discussed in the MTBs.
- Wilms tumor (n = 34) was the most commonly discussed cancer, followed by germ cell tumors (n = 13) and rhabdomyosarcoma (n = 12).
- 226 distinct management decisions were reached during the MTBs, of which 129 were subsequently implemented.
- Of these decisions, 15 resulted in changes in diagnosis and 53 were classified as major changes in management.
- Decisions on chemotherapy were the most likely to be successfully enacted (51/58), followed by radiotherapy (18/30) and surgery (12/21). Consults (16/40) and laboratory/imaging recommendations (21/53) were less likely to be carried out.

**Conclusions of the study**

- With a significant number of patients reviewed, new diagnoses made, and changes in management discussed, the MTBs are an important aspect of pediatric oncologic care at the UCI.
- However, the results also show that key improvements, specifically in the documentation and implementation of management decisions, are needed to improve the effectiveness of the MTB.
